# Enhancing Knowledge and InterProfessional care for Heart Failure (EKWIP-HF) in long-term care: a pilot study

**DOI:** 10.1186/s40814-017-0153-8

**Published:** 2017-07-06

**Authors:** George A. Heckman, Veronique M. Boscart, Kelsey Huson, Andrew Costa, Karen Harkness, John P. Hirdes, Paul Stolee, Robert S. McKelvie

**Affiliations:** 1 Research Institute for Aging, 250 Laurelwood Drive, Waterloo, Ontario N2J 0E2 Canada; 2 School of Public Health and Health Systems, University of Waterloo, 200 University Avenue West, Waterloo, Ontario N2L 3G1 Canada; 3 School of Health & Life Sciences and Community Services, Conestoga College Institute of Technology and Advanced Learning, 299 Doon Valley Dr, Kitchener, Ontario N2G 4M4 Canada; 4 Department of Clinical Epidemiology and Biostatistics and Medicine, McMaster University, 1280 Main St W, Hamilton, Ontario L8S 4L8 Canada; 5 Heart Failure and Cardiovascular Chronic Disease Management, Cardiac Care Network, 4100 Yonge St, North York, Ontario M2P 2B5 Canada; 6 Hamilton Health Sciences Corporation, 1200 Main St. West, Hamilton, Ontario L8N 3Z5 Canada; 7 McMaster University, 1280 Main St W, Hamilton, Ontario L8S 4L8 Canada; 8 Western University, London, Ontario Canada

**Keywords:** Nursing home, Heart failure, Interprofessional, Education

## Abstract

**Background:**

Heart failure (HF) affects 20% of long-term care (LTC) residents and is associated with significant morbidity, acute care visits, and mortality. Barriers to HF management are staff knowledge gaps and ineffective interprofessional (IP) communication. This pilot study assessed the acceptability, feasibility, and impact of an intervention to (1) improve HF knowledge; (2) improve IP communication; and (3) integrate improved knowledge and communication processes into work routines.

**Methods:**

The intervention provides multimodal IP education about HF in LTC, including specialist-supported bedside teaching. It was piloted on single units in two facilities. A mixed-methods repeated-measures approach was used to collect qualitative and quantitative process and outcome data at baseline and 6 months post-intervention.

**Results:**

Results were similar at both sites. Participants developed optimized IP communication to promote HF care. Results indicate a perceived increase in staff confidence and self-efficacy, strengthened assessment and clinical proficiency skills, and more effective IP collaboration. Staff deemed the intervention useful and feasible.

**Conclusions:**

This pilot study suggests that a novel intervention in which HF-specific knowledge is applied by LTC staff to improve IP collaboration in their own work place is acceptable and feasible and has a favourable preliminary impact on staff knowledge and IP communication.

## Background

Heart failure (HF) develops when the heart can no longer sustain sufficient output to meet the body’s metabolic demands [1]. Over 500,000 Canadians are affected by HF, most of whom are older adults [2]. HF affects 20% of long-term care (LTC) residents, in whom it is associated with significant morbidity and mortality, and accounts for a sizeable share of unplanned transfers to acute care hospitals [3–5]. Such transfers could potentially be avoided, and resident quality of life optimized, if effective HF care processes were in place in LTC homes [6–8]. Though guidelines endorse standard therapies for older patients, the management of HF in LTC homes does not often reflect guidelines due to concerns over diagnostic accuracy, polypharmacy, and skepticism about the benefits of HF therapies in frail seniors [5, 9–14].

Optimal HF management requires accurate diagnosis, appropriate treatment, monitoring, and symptom assessment, and tracking of fluid balance and weight [9–11]. Care delivered in an interprofessional (IP) chronic disease management framework has been shown to be highly effective at reducing acute care use and mortality among community-dwelling seniors with HF [9–11]. A few studies have shown that guideline-based HF management interventions in LTC can improve care quality and prevent hospitalization, though these were not designed with full stakeholder engagement nor directly addressed IP practice change, while others targeted less frail residents, relied on external resources, or focused on limited aspects of HF care [5, 15–23].

Our aim is to develop sustainable and effective IP HF care processes in LTC. Our initial work identified health service challenges faced by LTC residents with HF [4, 24–26]. Subsequent work showed that the two most important barriers to optimal HF management in LTC are knowledge gaps about HF among staff and ineffective IP communication [27–33]. Based on this work, we developed an intervention to improve HF care in LTC: Enhancing Knowledge and Interprofessional care for HF (EKWIP-HF). EKWIP-HF has three components: (1) improve staff knowledge about HF; (2) develop efficient IP communication processes to better manage HF in LTC; and (3) engage LTC staff to apply HF knowledge to the development and integration of IP communication processes into regular work routines. We conducted a pilot study to evaluate the acceptability and feasibility of the intervention.

## Methods

A mixed-methods repeated-measures design was used to collect qualitative (focus groups, interviews, observations) and quantitative (surveys, scales) process and outcome information to address the study objectives. EKWIP-HF was piloted over 6 months in a convenience sample of two units of two LTC homes in South Central Ontario, Canada. In each home, a unit was selected with input from facility administrators and nursing directors, one based on the high function of the team and the other because of a high proportion of convalescence residents with HF.

### Program description

EKWIP-HF has five phases (Table 1) sequenced based on our prior work emphasizing the importance to first address HF knowledge and IP communication among front-line staff, including Personal Support Workers (PSW), Registered Practical Nurses (RPNs), and Registered Nurses (RNs); physicians were thus excluded from the first three phases [33]. The first two phases took place in the first month. In phase 1, educational modules were delivered to all PSWs and nurses by HF/LTC specialists: GAH is a geriatrician specialized in HF care; VMB is a gerontological nurse; RSM is a cardiologist specialized in HF care. In addition to copies of the educational material, care planning tools identified in the literature were made available to staff, who were encouraged to appraise their clinical utility. These tools included the “ANEWLEAF” mnemonic, the “HF Zones” guidance, and the “Stop and Watch Early Warning Tool” from the Interventions to Reduce Acute Care Transfers (INTERACT) program for LTC, all of which were provided as laminated pocket cards and sheets (available from authors upon request) [34–36].

**Table 1:** Phases of EKWIP-HF pilot

Phase	Elements	Actions
Phase 1: address knowledge gaps Target: PSWs and RPNs Observers: MDs, RNs, administrators Month 1	Small-group interactive education sessions held at each LTC home	*Importance of HF*: basic physiology and impact on residents and health system *Clinical Skills*: recognize possible HF: -Classical and atypical symptoms (changes in cognition, mobility and function) -Edema and other basic symptoms and signs -Understand importance and meaning of weight changes -Understand course of HF, including acute and chronic aspects *Procedural skills*: -Rationale and methods for regular weights and reporting -Rationale for HF medications disease modifying vs. symptom control with diuretics -Understand role of the MDS 2.0 and Clinical Assessment Protocols
	Educational resources	PowerPoint of education material Pocket cards
Phase 2: develop communication processes for HF Target: PSWs and RPNs Observers: MDs, RNs, administrators Month 1	Workshop Develop/adapt processes for better communication between PSW and RPN	Review current communication processes and identify barriers and reasons for breakdown, framed in the context of HF (e.g. workload, staff scheduling, staff role). Develop/adapt communication processes focused on key episodes of HF care: shift change, physician rounds, new admissions, ad hoc identification of acute resident health deterioration, and measuring and tracking weights. Define required staff roles for the new processes (may be unique to each home). Define *process uptake indicators* to measure fidelity (e.g. communication logs for shift change and communication of weight changes, weight tracking tools)
Phase 3: implement communication processes and consolidate knowledge Target: PSWs and RPNs Observers: MDs, RNs, administrators Months 2 to 3	Communication process implementation and consolidation	Audit and feedback Audit mechanisms -Bi-weekly *on-site observations* by research assistant (shift change, work day) -Review of documentation of *process uptake indicators* -Weekly feedback from MDs, RNs and administrators about their observations Feedback: monthly meetings of PSWs and RPNs with research team to: -Review observations and identify potential changes to communication or documentation processes -Resolve knowledge and communication problems that have arisen Engage staff and encourage autonomy in conducting own audit and feedback reviews.
Phase 4: address knowledge gaps Target: MDs, RNs Month 3	Small-group interactive education sessions	Topics as in phase 1 (tailored to role), as well as: *Clinical skills:* physical assessment -Additional focus on volume assessment and determining “dry weight” *Procedural skills*: -Role of diagnostic testing -Appropriate prescribing, including adjusting diuretics based on weight changes -Discussing Advance Care Planning
Phase 5: full interprofessional integration Target: all clinical staff Observers: administrators, residents/families Months 3–6	Bedside teaching (six monthly sessions)	Sessions to take place during regular LTC physician visits and -Will take no more than 30 min -Include physician, nurses, PSW, and other staff assigned to a resident with HF With resident consent, bedside clinical assessments will be conducted with the LTC team present and participating. Case will be discussed and care management and communications plan will be developed by the team. Sessions will be facilitated by the expert clinicians, with the aim to promote greater engagement and autonomy by the LTC team.
	Case conferences (six monthly sessions)	Conferences to take place during regular LTC physician visits and will consist of a discussion of a resident’s case with HF. Initially, conferences will be facilitated by the HF and LTC expert clinicians, with the aim to promote greater engagement and autonomy by the LTC team.

In phase 2, an IP working group (PSWs, RPNs, RNs), reflecting staff mix and both day and night shifts, was established on each unit. Volunteers from phase 1 were recruited to the working groups, and using a snowball technique identified other colleagues whom they felt could make effective contributions to the group. Each working group attended a day-long workshop consisting of a facilitated discussion to first identify existing communication processes on their units and then apply HF management principles to refine or develop new processes to guide care during five key care episodes identified in our previous work: (1) new admissions; (2) shift changes; (3) physician rounds; (4) weight monitoring; and (5) acute resident instability [33].

Phase 3 took place in month 2, in which working groups implemented communication and documentation processes on each pilot unit. Following implementation of these processes, a trained research assistant (RA) visited each LTC home bi-weekly to conduct on-site observations and review adherence to processes. Regular meetings were held with members of the working groups and the research team to review observations, identify potential changes to IP processes, and resolve any knowledge and communication problems.

The last two phases took place in the next 4 months. In phase 4, LTC physicians attended education sessions which focused on clinical skills (e.g. physical assessment, volume assessment, determining “dry weight”) and procedural skills (e.g. role of diagnostic testing, appropriate prescribing, Advance Care Planning). In phase 5, the HF/LTC specialists facilitated bi-weekly bedside sessions during regular physician rounds with all available members of the working groups. Case conferences also took place during regular LTC physician visits to discuss cases of residents with HF. Bedside sessions and conferences were initially conducted by the HF/LTC expert clinicians, with working group members subsequently encouraged to take on this role.

### Data collection

We examined the utility of five validated questionnaires to assess the impact of the intervention on HF knowledge and IP communication. As no HF knowledge surveys specific to the LTC setting exist, we chose two community-based HF knowledge surveys in order to best understand their suitability for this and subsequent projects.

1. *Dutch HF Knowledge Scale*: This is a 15-item self-reported questionnaire. Multiple-choice questions assess knowledge about HF and symptom recognition, medication, fluid restriction, diet, and exercise [37]. Each question has three possible answers, with one being correct. The scale is a reliable and valid tool to assess HF knowledge among community-dwelling patients and their caregivers and was thus chosen as a potentially useful measure given the high proportion of PSWs in LTC.
2. *Nurses Knowledge of Heart Failure Education Principles* (*NKHFEP*): This is a 20-item, true/yes or false/no questionnaire developed to assess the abilities of acute care and community-based nurses to educate patients and caregivers about HF self-management principles [38]. The scale consists of items related to signs and symptoms of worsening condition, fluids/weight, diet, exercise, and medications. Face and content validity and test-retest reliability have been established.
3. *The Bridge Project surveys*: These surveys were specifically developed to measure LTC staff confidence in caring for residents with HF [19]. Items were chosen based on recommended HF management practices. A Likert scale is used to assess confidence with 1 indicating “no confidence” and 5 being “strongly confident”. Scores range from 0 to 45 for PSWs and 50 for nurses.
4. *Individualized Care Communication Subscale* (*ICCSS*): This is a brief 10-item scale measuring IP communication between LTC caregivers, including front line staff and supervisors [39]. Items were developed through an expert panel, and face and content validity were established in the care of persons with dementia. Items are rated on a 4-point scale.
5. *Interprofessional Socialization and Valuing Scale* (*ISVS*): This is a 24-item self-reported scale developed to evaluate the shift towards collaborative care approaches in various health care settings [40]. The version used assesses the quality of IP relationships using a 5-point Likert scale (1 = strongly agree; 5 = strongly disagree), with a “not applicable” option. The validity and reliability of the ISVS has been established.

Additional assessment of the feasibility, acceptability, and preliminary impact of the intervention was conducted through regular on-site observations and focus groups and interviews with working group members [41]. Trained RAs visited both LTC homes weekly to observe and keep detailed field notes related to adherence to and quality of communication processes. Field notes were also taken independently by the RAs during each meeting, education session, IP workshop and bedside session. The RAs were unaffiliated with either the LTC homes, their staff, or their residents. The interviews and focus groups addressed staff perspectives of the impact of the intervention on HF knowledge and the quality of communication among the LTC staff. Purposive sampling was used to intentionally recruit those working group members that actively participated in all phases of EKWIP-HF for interviews [42].

### Data analysis

Quantitative data were analysed to assess changes in LTC staff, HF knowledge, self-efficacy, and IP collaboration. Bivariate descriptive statistics were used to characterize care providers. Paired *t* tests were used to assess statistical significance of any pre-post changes. All analyses were performed using SAS version 9.4 for Windows (SAS Institute, Inc., Cary, NC). Qualitative data were transcribed from digital recordings and organized. Using thematic content analysis, data were carefully read to identify underlying concepts and concept clusters (NVIVO 10.0) [43–45]. Two investigators analysed the data separately and developed major emerging themes. Data analyses were conducted in an iterative manner until consensus within the research team was reached. Member checking was undertaken by presenting established themes and gathering feedback from individual working group members in order to assess the validity of findings and ensure that data were interpreted correctly [46]. Use of several methods ensured the credibility of the findings. Data triangulation occurred data were collected from multiple sources (focus groups, interviews, and RA field notes). After coding was completed, data were organized according to major categories identified. Next, subcategories were identified reflecting narrower topical areas within major categories. Once the research team collaboratively reviewed and agreed upon the identified subcategories, major themes that emerged from the data were identified and described.

## Results

### Demographic data

Unit 1 housed 32 residents in a private facility, was staffed by eight PSWs and two RPNs, and was supported by one RN and attending MDs. Unit 2, a convalescent care unit in a municipal facility, housed 25 residents, was staffed by seven PSWs and four RPNs, and was supported by four RNs and one MD within a 24-h period. Table 2 describes the characteristics of the working group participants (8 on unit 1, 19 on unit 2). The majority were female PSWs between 35 and 44 years old. Over 40% had over 10 years of experience working in LTC.

**Table 2:** Characteristics of LTC working group participants

Baseline characteristic	All participants (*n* = 27)
% Female	88.2%
Age	
<35	2 (7.4%)
35–44	12 (44.4%)
45–54	8 (29.6%)
55–65	5 (18.5%)
Professional designation	
PSW	16 (59.3%)
RPN (including 1 student)	8 (29.6%)
RN	3 (11.1%)
Employment status	
Full time	20 (74.1%)
Part time	6 (22.2%)
Student placement	1 (3.7%)
Shift schedule	
Day	19 (70.4%)
Evening	6 (22.2%)
Day and evening	2 (7.4%)
Education level	
Certificate	12 (44.4%)
Diploma	10 (37.0%)
Bachelor’s degree	2 (7.4%)
Other^a^	3 (11.1%)
Years worked in long-term care	
1–3	2 (7.4%)
4–10	14 (52.0%)
≥11	11 (40.7%)
Years worked at site	
0–3	5 (22.2%)
4–10	14 (52.0%)
≥11	7 (25.9%)

^a^Other education level included working towards a diploma and educated abroad

### HF knowledge, self-efficacy, and IP communication

Baseline and post-intervention scores on the five surveys are presented in Table 3. Scores on HF knowledge scales showed improvement, though only the Bridge scales showed statistically significant improvement. There were no significant changes in IP scores. Due to turnover and shift work variability, not all working group members completed both baseline and follow-up surveys. Change in scores among completers of both assessments is shown in Table 4. There were no significant differences in baseline scores between completers of both surveys, compared to completers of the baseline survey only, though the former tended to achieve higher baseline knowledge scores (data not shown).

**Table 3:** Overall scores from all respondents

Scale	Baseline			Post-intervention			*p*
	*N*	Mean (%)	Std dev (%)	*N*	Mean (%)	Std dev (%)	
ICCSS	17	72.4	9.2	16	75.0	16.0	0.26
ISVS	16	80.5	12.8	17	79.3	20.1	0.83
Dutch	25	83.5	13.1	17	88.6	12.0	0.31
Bridge	25	77.2	10.8	16	85.9	8.2	0.035
NKHFEP	24	67.1	12.1	16	69.7	8.7	0.48

All scores standardized to percent. Bridge scores for nurses and PSWs were collapsed into an overall score because of small sample. *ICCSS* Individualized Care Communication Subscale, *ISVS* Interprofessional Socialization and Valuing Scale, *Dutch* Dutch HF Knowledge Scales, *Bridge* Bridge Project Surveys, *NKHFEP* Nurses Knowledge of Heart Failure Education Principles

**Table 4:** Change from baseline among baseline and post-intervention respondents

Scale	Number	Mean change	95% confidence interval
ICCSS	10	−4.40	−12.65, 3.85
ISVS	10	1.15	−12.79, 10.49
Dutch	16	4.48	−4.57, 13.52
Bridge	15	8.00	0.656, 15.34
NKHFEP	14	3.21	−6.37, 12.80

All scores standardized to percent. Bridge scores for nurses and PSWs were collapsed into an overall score because of small sample. *ICCSS* Individualized Care Communication Subscale, *ISVS* Interprofessional Socialization and Valuing Scale, *Dutch* Dutch HF Knowledge Scales, *Bridge* Bridge Project Surveys, *NKHFEP* Nurses Knowledge of Heart Failure Education Principles

### Acceptability, fidelity, and feedback

Observations and interviews indicated that the intervention led to a perceived increase in staff knowledge and confidence about HF assessment, greater proficiency in clinical skills, and more effective IP communication and collaboration. Working group members deemed the intervention useful and feasible, highlighting their appreciation of the educational content, the bedside sessions, and preferences for specific tools. In addition, working group members rebranded themselves as the “Core Heart Team” (CHT) for their home. This term shall henceforth be used to designate the working groups.

#### Knowledge

CHT members recognized that their involvement with EKWIP-HF improved their knowledge and clinical assessment skills. Many emphasized that the education increased their awareness that symptoms of HF in LTC residents can be non-specific. One member discussed a cognitive shift from associating non-specific clinical features with dementia only to now considering alternate diagnoses:

“This thing [EKWIP-HF] has really trained us with that knowledge. It has helped us think about things in a bigger perspective. A lot of symptoms, like the delirium or the restlessness at night, I would have just thought, like before this all started, ‘it’s dementia, it’s dementia,’ and I think that is a mentality in [our homes and probably] long-term care in general, right? It’s such a broad statement but now, it’s like ‘ok…it might not be. Let’s look into this.’”—PSW

The education sessions prepared CHTs, particularly PSWs, to participate in clinical assessments of residents. Active involvement in the educational components of EKWIP-HF (education modules, bedside sessions, case conferences) increased members’ ability to properly identify HF cases, as described by a CHT member:

“We have been more vigilant. I think we have been a little better at identifying cases of heart failure that we have.”—RPN

Increased awareness of HF was a major theme among the CHTs at both LTC sites. Members were more aware of HF signs and symptoms, diagnoses, treatment, and care processes as a result of EKWIP-HF. One member expressed this and the importance of working together as an IP team:

“This has really opened up my mind too, my eyes, with the admission process as well…I’m looking for if there is a heart failure diagnosis. I want to find someone now because then we can talk about this as the team, soon to be experts.”—PSW

Many CHT members emphasized how EKWIP-HF facilitated new and more opportunities for knowledge exchange among the various LTC staff roles. Members felt comfortable that they could learn from one another. In particular, many realized the opportunities of learning from PSWs. One physician said:

“There’s a lot of bottom-up education that can go on for the RNs and physicians, and I’ll speak for the physicians in particular, because if they know that the PSWs are more mindful of changes in weight, edema…they are going to be more aware of how their patients are monitored and perhaps look for more things in the progress notes.”—MD

#### IP communication and collaboration

CHT members recognized that not only did EKWIP-HF facilitate better IP communication among team members but also better communication facilitated better care more broadly, and not just for HF. One member explained:

“It’s the process that this whole study has put in place for us. It has not only given us more knowledge about heart failure…but it has made us and helped us communicate better as a team. ‘If something is a little off, let’s talk about it. Let’s report it’…So it doesn’t just end here. You can apply it to anything really. It’s really not just heart failure.”—PSW

CHT members valued the involvement of different LTC staff roles within the team. As described by one RPN, the expression of multiple perspectives was encouraged through greater collaboration:

“I really enjoyed the input, and you guys [other CHT members] had a totally different aspect of things that we don’t see and it really opened my eyes to a lot of things that I would never think of. Basically, I prefer everybody together.”—RPN

Another benefit of enhanced collaboration mentioned was that of mutual learning. One physician noted:

“I think it can be applied to a number of other things and as a physician who really doesn’t work that closely with PSWs, my question at the end of it is how can I tap into their knowledge a bit more than I presently do because they are valuable bedside observers.”—MD

#### Impact on residents and family members

All CHT members interviewed were of the opinion that residents and family members benefited from the IP communication processes and attitudes promoted by EKWIP-HF. Referring specifically to the bedside rounds, one physician said:

“The real benefit was the patient and family by the bedside. We saw a variety of patients when we did rounds but I think they all really felt they were receiving special attention and they learned from the discussion going on at the bedside as well…and they felt that they were made privileged to things that are more often talked outside the door or out of their ear shot.”—MD

Increased IP communication and collaboration led to the identification of residents with potential HF through the sharing of observations made by individual CHT members and ensuing discussions. One member described how several PSWs came together to discuss their observations of a newly admitted resident with no known prior diagnosis of HF, concluded together that HF was a possibility, and brought their concerns to the nurse and attending physician. The team and physician assessed the resident and confirmed the diagnosis:

“They increased her Lasix and after 3 or 4 days she felt so much more comfortable, and the coolest thing was [HF/LTC specialist] had said, ‘this resident […] there is a very, very good chance that she will not die of heart failure, which is a terrible, slow death, right? It will probably be from something else but you guys have prevented that from happening, and it’s holy smokes…that was amazing.”—PSW

The quotation above also highlights not only the importance of communication from PSWs to physicians but also the importance of positive feedback in engaging staff.

#### Feedback on tools and scales

Most CHT members preferred the ANEWLEAF pocket card. Cards were posted throughout both units, as they were considered valuable for everyone. One physician explained:

“It may not hurt to post the ANEWLEAF pocket cards in the residents’ rooms so that family, residents, and front line workers can recognize and act if necessary.”—MD

Members also found ANEWLEAF to be a useful guide for clinical assessments and facilitating communication. One nurse described how the card was used during bedside rounds:

“I wasn’t too sure how to do the format of the rounds but then I thought I might as well use the card…to discuss her - that would make sense. So, that’s what we did.”—RN

Several participants suggested that the “HF Zones” guidance was also a useful reference for LTC staff, and others perceived its value as a self-management tool to engage residents and families. One CHT member described using the tool:

“Just having that up on the board…it’s almost the 3-step process. One is identifying the residents based on the information we’ve learned. Two is the communication paired with the assessment; and then three is ‘okay, now I’m going to refer to the zones.’ So, just in that quick 3-step process, you’ve come up with something, and then the next step obviously is to get to the doc.”—PSW

#### Acceptability

All CHT members interviewed considered all components of EKWIP-HF as essential for its success. One said:

“Now that we’re at the tail end of it, I can go back and just see the importance of the components – of all the components. I think the education sessions, the workshop that we did - the eight hour workshop. I think that was very cool.”—PSW

Some members specified certain components of EKWIP-HF as particularly beneficial. Bedside rounds were considered an essential “team building” component of the intervention, as emphasized by this physician:

“I thought it was useful. I also thought it was good to have everyone in the same room. I mean, I think the biggest thing is honestly the team building component to it.”—MD

#### Fidelity

Fidelity of the IP communication and documentation processes varied. Participants perceived some processes to be easier to implement than others, as stated by one CHT member:

“Just to kind of review these interventions then – some got done, some didn’t obviously. Some are easier to do than others.”—RN

As a result, both teams requested half-day refresher workshops which were scheduled four months into the intervention.

Tracking daily weights of residents with HF was considered feasible, though burdensome to residents. Though able to conduct daily weights, both teams noted, in the context of increased vigilance by CHTs, that residents with HF could be weighed less often, depending on the presence of acute changes. Furthermore, it was suggested that weighing frequency could be adapted based on resident stability. One member explained:

“At the beginning, we used to weigh people with the diagnosis daily. It was no changes for the longest time because they were compensating. They were being treated properly. So, since there were no red flags, which you have to keep such close monitoring, we changed it. So, it is relative. Ideally, it has to be at least weekly, sometimes bi-weekly.”—PSW

“For the sake of your project, we were doing it daily on patients with CHF but decided that it was too much for various reasons. Too much work for no reason…then we changed it to weekly unless identified as an issue where we would have to go to 2 or 3 times weekly; still not as far as daily.”—RN

#### Feedback on EKWIP-HF

CHT members offered feedback to enhance various aspects of the intervention. Greater variety in educational modalities was suggested by several participants. One member suggested:

“If you added different aspects of learning…the video for the visual or even with a case study, ask for a specific resident that has CHF so that everybody can apply the knowledge to something that they already know…and you can throw some humour in there, some nice pictures to catch peoples’ interest…and chocolate, of course.”—RN

Several members felt that more structured guidelines to guide the conduct of initial IP bedside rounds would be helpful. One nurse recommended:

“Since those rounds are so important, maybe a stricter format just so people have an expectation of what their role is in it or how to go about. I think for the first couple…it was kind of painful I thought. Nobody really knew and it was the first time they tried to apply the knowledge.”—RN

Another also proposed a more structured approach to the IP bedside rounds:

“I had a hard time but I only went to 2 of the bedside rounds. The first one I walked in and it looked like nobody had no plan, no direction, nothing! We should have gotten together before. It’s just my opinion, that ‘this is what we’re looking for. I need you to be responsible for this’…because the second time was amazing! I felt like I paid something to go somewhere to watch and learn. But the very first one, wow, ‘what’s going on here?’…I know it was all new for us too, and I only went to 2 and wow! What a difference!”—RPN

Many CHT members noted the need for leadership within the team as crucial for successful implementation of EKWIP-HF. One physician commented:

“I think you need a quarterback. I mean, you need a quarterback to say ‘we need to get everyone together and meet on this day’ and everything else. Starting from a functional standpoint, you need someone to coordinate everything. But to me, the whole project in essence was about team… team building and team empowerment.”—MD

However, it was agreed that a team leadership style was required that promotes empowerment and accountability among team members. One nurse stated:

“I think an important part about the leadership too though is empowering the rest of the team…getting their opinions and making them feel they’re a valuable part of the project.”—RN.

## Discussion

The results of this pilot study suggest that EKWIP-HF was acceptable and feasible. The results, based mainly on the qualitative feedback, also suggest that the intervention has the potential to improve HF knowledge and communication among staff and can lead to the detection of HF among residents, even in those without a prior diagnosis. Finally, the pilot study yielded helpful information on how to improve the implementation of the intervention.

While scores on the scales designed to measure IP communication did not change significantly, data from the interviews suggest important improvements in IP communication and attitudes between nurses, PSWs, and physicians. The limited change measured by the scales may reflect several factors. This was a pilot study, not powered to identify clinically meaningful change. The relatively high baseline IP scores may reflect high functioning teams in the LTC homes that volunteered to participate in the pilot. Furthermore, a recent systematic review suggests that most instruments intended to measure IP collaboration are not well designed to identify change resulting from IP educational interventions [47].

HF knowledge improved, as shown by the qualitative data and Bridge scores. Scores on the other instruments, while showing positive trends, did not change significantly. Baseline scores on the Dutch HF Knowledge scale were similar to other published studies, while those on the NKHFEP were lower [37, 48]. However, both instruments were designed for nurses providing care or education to community-dwelling patients with HF, and the latter includes questions on dietary choices and contraindicated medications, issues beyond the practice scope of PSWs and RPNs in LTC. Indeed, the qualitative data suggest that key learnings as a result of EKWIP-HF were associated with greater understanding that HF in frail LTC residents can present atypically (e.g. behavioural symptoms), and thus, instruments specific to knowledge about HF in LTC are required. Improvements in Bridge scores suggest that the intervention also led to greater confidence and self-efficacy, which is more predictive of performance than satisfaction with an education session or perceived learning [49, 50]. These results also suggest the need to develop instruments to measure HF knowledge and that are specific to LTC.

Study participants provided important feedback on the intervention. The project provided support for the use of the “ANEWLEAF” and “HF Zones” tools, which were identified as particularly helpful for resident assessment and supporting care planning. Improvements were recommended to make the educational sessions more interactive (e.g. discussing actual residents who may have HF) and to provide teams with a standardized template to conduct the bedside sessions.

A number of limitations must be noted. The pilot study was small and was conducted on self-selected units with motivated volunteer staff. Sustainability of the intervention, and its wider dissemination, both within an entire facility and beyond to other facilities, remains to be demonstrated. The impact of the intervention over the longer term on resident quality of life, acute care utilization, end-of-life care, and LTC staff job satisfaction also needs to be determined. In particular, it will be important to establish how the Core Heart Team approach, in contrast to models based on individual champions, is able to withstand the impact of personnel turnover, which is common in LTC. Ways to mobilize community HF specialists and HF management programs to provide ongoing clinical and capacity-building support to LTC HF initiatives are required.

## Conclusions

The results of this pilot study provide preliminary evidence that the EKWIP-HF intervention is both acceptable to LTC staff and feasible to carry out. The study yielded important information on how to improve and streamline the intervention and assess its impact more accurately. Further, the results also suggest that this novel intervention, in which LTC staff learn how to apply HF-specific knowledge to improve IP collaboration in their own work place, also has the potential to improve resident outcomes. The next steps in our work are to develop a framework to ensure the sustainability and dissemination of the intervention. We will base this work on the concepts of sustainability recently developed and validated by Fleiszer et al., in which outcome and process measures must be embedded into routine practice (*routinization*) so that data (*observability of benefits*) can support intervention sustainability and innovation (*development*) [51, 52]. We will then be in a position to evaluate the entire EKWIP-HF and sustainability bundle in a larger pilot study and subsequently in a clinical trial.

## Abbreviations

CHT: Core Heart Team
EKWIP-HF: Enhancing Knowledge and Interprofessional care for Heart Failure
HF: Heart failure
ICCSS: Individualized Care Communication Subscale
INTERACT: Interventions to Reduce Acute Care Transfers
IP: Interprofessional
ISVS: Interprofessional Socialization and Valuing Scale
LTC: Long-term care
MD: Medical Doctor
MDS: Minimum Data Set
NKHFEP: Nurses Knowledge of Heart Failure Education Principles
PSW: Personal Support Worker
RA: Research Assistant
RN: Registered Nurse
RPNs: Registered Practical Nurses
